# Predictors of breastfeeding self‐efficacy among women attending an urban postnatal clinic, Uganda

**DOI:** 10.1002/nop2.257

**Published:** 2019-03-21

**Authors:** Joyce Nankumbi, Ashely Atwiine Mukama, Tom Denis Ngabirano

**Affiliations:** ^1^ Department of Nursing Makerere University Kampala Uganda; ^2^ Gulu Independent Hospital Gulu Uganda

**Keywords:** breastfeeding, midwifery, nursing, postnatal women, self‐efficacy, Uganda

## Abstract

**Aim:**

The aim of this study was to determine the factors associated with breastfeeding self‐efficacy among postnatal women in Kampala, Uganda.

**Methods:**

This was a descriptive cross‐sectional study that was conducted among women attending a postnatal clinic at a teaching hospital in Kampala. Three hundred and eighty‐four postnatal women were randomly selected to respond to an interviewer‐administered questionnaire. We used the Breastfeeding Self‐Efficacy scale (BFSES) to assesses breastfeeding self‐efficacy (BFSE). Descriptive statistics and percentages were used to summarize the findings. Bivariate and multivariate logistic regressions were used to determine predictors of BFSE.

**Results:**

Participants had a mean BFSE score of 48.65. The 14 item BFSES consistently measured breastfeeding confidence with a Cronbach's alpha of 0.89. About six in 10 women (60.2%) had high BFSE, the rest (39.8%) had low BFSE. Having a partner (adjusted odds ratio (aOR): 13, 95% CI 3.46–15) and receiving breastfeeding support from health workers (aOR: 4.45, 95% CI: 1.95–6.12) were significantly associated with BFSE.

**Conclusion:**

A notable number of mothers had a low BFSE. Health workers should support breastfeeding mothers to achieve the desired exclusive breastfeeding levels.

**Relevance to clinical practice:**

The findings of the study provide a direction for midwives in maternity care in educating and supporting women about breastfeeding for the improvement of exclusive breastfeeding rates thus realization of benefits of exclusive breastfeeding.

## 1 INTRODUCTION AND BACKGROUND

World Health Organization (WHO) recommends that mothers should exclusively breastfeed for 6 months and continue breastfeeding with other feeds up to 2 years and beyond (WHO, 2005). On the other hand, the benefits of breastfeeding both to the mother and the baby are clearly documented (Black et al., 2008; Rollins et al., 2016; Wambach & Riordan, 2014). Research has shown that children who are breastfeed have low risks of suffering from diarrhoeal diseases and other illnesses (Richard et al., 2018). Mothers who breastfeed their babies are able to bond with their infant, loss of the pre‐pregnancy weight, child spacing by lactation amenorrhoea and their uterus contracts well (Kair, Flaherman, Flaherman, Newby, & Colaizy, 2015). Mothers should be able to initiate breastfeeding within the first hour after delivery, exclusively breastfeed for 6 months and then continue breastfeeding until 2 years or more (World Health Organization, 2013). There are various factors that determine successful breastfeeding among mothers. Among these include social support, education level, age, maternal confidence to breastfeed and previous experiences (Brown, 2014). Breastfeeding self‐efficacy (BFSE) which is a mother's confidence in her ability to breastfeed has been found to be highly predictive of breastfeeding behaviours (Tokat, Okumuş, Okumuş, & Dennis, 2010). A relationship has been demonstrated between BFSE and exclusive breastfeeding (Alus Tokat, Serçekuş, Serçekuş, Yenal, & Okumuş, 2015). Maternal BFSE has been highlighted as an important factor for improving breastfeeding outcomes (Keemer, 2013; Nursan, Dilek, Dilek, & Sevin, 2014; Otsuka et al., 2014; Tuthill, McGrath, Graber, Cusson, & Young, 2015).

In Uganda, despite the universal knowledge on breastfeeding, Uganda Bureau of Statistics (UBOS) reported an exclusive breastfeeding rate of 66%, yet 98% of the infants are initiated to breastfeeding (UBOS, ). Premature cessation of breastfeeding is postulated to be common among primiparas, mothers with HIV/AIDS, working mothers and women with low confidence to breastfeed. The breastfeeding rates indicate that mothers and infants are not receiving the maximum health benefits.

Whereas measuring of the BFSE of mothers using the BFSE scale‐short form (BFSES‐SF) has been done in many developed countries to understand the relationship between breastfeeding and BFSE, there is barely any documentation on BFSE and its predictors yet women can benefit from education during the antenatal period through sessions by the midwives and other healthcare professionals in promotion of (Küçükoğlu & Çelebioğlu, 2014; Zhu, Chan, Chan, Zhou, Ye, & He, 2014).

Breastfeeding self‐efficacy has been documented as a strong determinant of breastfeeding behaviour of women. Women with high BFSE are likely to breastfeed their infants compared with someone with low confidence. Studies elsewhere have also reported that BFSE is associated with maternal age, economic status, method of delivery and maternal demographic factors mood, low birth weight of babies, hospital support (Brown et al., 2013; Henshaw, Fried, Fried, Siskind, Newhouse, & Cooper, 2015; Nursan et al., 2014; Otsuka et al., 2014; Zhu et al., 2014). Therefore, this study aimed at determining factors associated with BFSE.

## 2 METHODS AND MATERIALS

### 2.1 Study design and setting

This cross‐sectional study was conducted to determine factors associated with BFSE of postnatal mothers. The study was carried out on postnatal units in a teaching hospital in Kampala, Uganda. The facility is one of the largest hospitals in the country offering maternal and child health services including antenatal, postnatal and special care for new born babies. The hospital has an official bed capacity of 1,790 beds. The postnatal section of this Hospital admits an average 100 mothers per day.

### 2.2 Study population and sample size

The study population included women who had delivered either normally or by caesarean birth. The study included women who voluntarily consented to participate in the study. Women who had birth complications and those who were too sick to participate in the study were excluded. We used Epi‐info version 7 to calculate the samples size based on a consideration of a two‐tailed significance level, an (alpha) of 0.05, an expected frequency of low BFSE of 50% and a 95% confidence level. Based on these considerations, we enrolled a total of 384 postnatal mothers.

### 2.3 Measurement of variables

#### 2.3.1 Breastfeeding self‐efficacy

We conceptualized BFSE as the mother's confidence in her ability to breastfeed her baby. This conceptualization was based on the theorization of Dennis in his BFSE theory (Dennis, 1999). Based on this theory, Dennis and Faux developed the BFSE (Dennis & Faux, 1999) which was tested among 130 Canadian women. The original 43 item version of BFSE has since been reduced to 14 items through rigorous methodological studies and found to be valid and reliable in China (Dai and Dennis, 2003), in Poland (Wutke & Dennis, 2007) and in Australia (Creedy et al., 2003). In the present study, BFSE was operationalized as a summated score on the 14‐item version. The scale is anchored on a 5‐point liker scale where 1 indicates not at all confident and 5 indicates very confident. The total score on this scale ranges from 14–70 and higher scores correspond to higher levels of breastfeeding confidence. The internal consistency of the BFSES in this study was 0.89, with a Median score of 52. Participants with scores equal to or more than 50 were considered to have higher BFSE, and those with scores below 50 were considered to have low BFSE (Wutke & Dennis, 2007. In addition, we solicited information about; age, marital status, tribe, level of education, employment status, average monthly income, parity, social support, previous breastfeeding experience and health facility related variables form each participant.

### 2.4 Sampling and data collection procedures

We randomly selected the participants from the postnatal clinic by writing numbers corresponding to total number of mothers admitted on the ward on pieces of paper. The pieces of paper were then placed in the containers and mixed thoroughly. Twenty pieces of paper were then picked without replacement; mothers corresponding to the picked pieces of paper were then approached and asked to participate in the study. Mother who accepted to participate in the study were then provided with detailed information, those who consented were then asked to respond to questions about their breastfeeding practices, social demographics and BFSE. Data collection was conducted in one of the rooms at the ward by two trained research assistants.

### 2.5 Data management and analysis

We conducted data analysis using SPSS version 23 statistical software. The levels of BFSE were computed by summing the 14 items of the BFSES. As proposed by (Wutke & Dennis, 2007), we categorized mothers with scores of 50 or more as having high BFSE and those with scores <50 as having low BFSE. We then conducted bivariate analysis and generated crude odds ratios with corresponding 95% confidence intervals (CI). Following bivariate analysis, variables with *p*‐values of 0.2 or more and those that we deemed plausible were considered for the multivariate binary logistic regression model. The significance level was set at 0.05 and all *p*‐values were two tailed.

### 2.6 Ethical considerations

Research Ethics committee approval was sought and secured from Makerere University School of Health Sciences Ethics Review Committee (SHSREC REF 2015‐047) and administrative approval from the hospital. Permission was also obtained from the postnatal unit head nurses. Written informed consent was also obtained from the study participants after provision of information about the study. Participants were assured of confidentiality and privacy by assigning them with numbers instead of names, and it was indeed maintained throughout the study. Participants had the liberty to withdraw from the study at any time, and this did not affect their access to the services at the clinic.

## 3 RESULTS

### 3.1 Characteristics of the study participants

The mean age of the participants was 26.3 (*SD*: 6.16) years with a range of 16–42 years. Nearly one half of the participants 183 (47.7%) had obtained secondary education. Slightly more than one half of the participants 207 (53.9) had delivered their babies by spontaneous vaginal delivery. The mean age of the babies was 2 (*SD*: 1.26) days. Most of the mothers 245 (63.8%) had delivered baby boys. The mean weight of the babies was 3.29 ± 0.72 kg (Table 1).

**Table 1:** Socio‐demographic characteristics of postpartum mothers

Characteristics variable	Frequency	Percentage
Age group		
16–25	183	47.7
26–35	168	43.8
>36	33	8.6
Marital status		
Single	46	12
Married	330	85.9
Widowed	8	2.1
Level of education		
None	13	3.4
Primary	123	32
Secondary	183	47.7
Higher institution	65	16.9
Mothers average monthly income		
15,000–150,000	74	19.3
>150,000	79	20.6
No monthly income	231	60.2
Social support		
Yes	294	76.6
No	90	23.4
Method of delivery		
Spontaneous vaginal delivery	207	53.9
Caesarean section	177	46
Age of baby (days)		
1–3	253	65.9
4–6	113	29.4
>7	18	4.7
Birth weight of baby(kg)		
0.75–2.50	41	10.7
>2.50	343	89.3
Breastfeeding experience		
Yes	254	66.1
No	130	33.9

### 3.2 Breastfeeding self‐efficacy of the postnatal mothers

The BFSE of the postnatal mothers was determined using the 14‐item short form of the BFSES. The items of this scale in the present study were highly consistent with a Cronbach's *α* coefficient of 0.89. The item mean scores ranged from 3.12 for item number two “I can breastfeed my baby without formula” to 3.93 for item number one “I can determine if my baby is getting enough milk.” The mean scores and standard deviations for the other items are presented in Table 2. Overall, the mean BFSES of postnatal mothers was 48.65 ± 8.36 with a median score of 52. The scores for the other items are presented in Table 2. Based on the cut‐off score of 50 which was proposed by Wutke and Dennis (2007), most postnatal mothers 231 (60.2%) had high BFSE and the rest 153 (39.8%) had low BFSE.

**Table 2:** Participants mean and standard deviations on the 14 items of the Breastfeeding Self‐Efficacy scale

Item	Mean score	*SD*
1. Determine if my baby is getting enough milk	3.93	0.67
2. Successful cope with breastfeeding like I have with other challenging tasks	3.47	1.08
3. Breastfeed my baby without a formula as a supplement	3.12	1.09
4. Ensure my baby is properly latched on for the whole feed	3.73	0.82
5. Manage the breastfeeding situation to my satisfaction	3.59	0.87
6. Manage to breastfeed even if baby is crying	3.59	0.92
7. Keep wanting to breastfeed	3.41	1.07
8. Comfortably breastfeed with my family members present	3.69	0.99
9. Be satisfied with my breastfeeding experience	3.73	1.22
10. Deal with the fact that breastfeeding can be time consuming	3.41	0.91
11. Finish feeding my baby on one breast before switching to another	2.41	0.87
12. Continue to breastfeed to breastfeed my baby for every feed	3.48	0.96
13. Manage to keep up with my baby's breastfeeding demands	3.48	0.92
14. Tell when my baby has finished breastfeeding	3.65	0.81

### 3.3 Factors associated with breastfeeding self‐efficacy

Mothers (231) who scored 50 of more on the BFSES were considered to have high BFSE, and those (153) who scored <50 were considered to have low self‐efficacy (Wutke & Dennis, 2007). Based on this categorization, we conducted bivariate and multivariate logistic regression analyses to identify factors associated with high BFSE. Factors that were significantly associated with high BFSE at bivariate analysis included having delivered by spontaneous vaginal delivery (crudes odds ratio (cOR): 1.79, 95% CI: 1.18–2.71), having no salaried employment (cOR: 2.84, 95% CI: 1.71–4.71), having breastfeeding experience (cOR: 1.63, 95% CI: 1.06–2.51), having delivered more than 2 days prior to the interview (cOR: 2.04, 95% CI: 1.29–3.20), having a partner (cOR: 8.92, 95% CI: 4.32–18.4) and having received breastfeeding support from health workers (cOR: 1.94, 95% CI: 1.20–3.12). At multivariate analysis, mothers who had partners had higher odds of having high BFSE compare to those who had no partners (adjusted odds ratio (aOR): 13, 95% CI: 3.46–23.11). In addition, mothers who received breastfeeding support from health workers had higher odds of having high BFSE compared with those who did not receive breastfeeding support from health workers (aOR: 4.45 95% CI 1.95–6.12). Details of both bivariate and multivariate analyses are presented in Table 3.

**Table 3:** Bivariate and multivariate logistic regression of predictors of high breastfeeding self‐efficacy

Variable	High BFSE	Low BFSE	OR	CI	*p*‐value	aOR	CI	*p*‐value
	*N* = 231	*N* = 153						
Mode of delivery								
SVD	119	57	1.79	1.18–2.716	0.006	1.06	0.36–3.12	0.91
C/section	112	96	1.00					
Age (years)								
≤26 years	104	83	0.69	0.45–1.04	0.07	1.43	0.70–2.92	0.31
26 or more	127	70	1.00					
Relationship								
Partner	221	109	8.92	4.32–18.40	<0.001	13	3.46–23.11	<0.001^a^
No partner	10	44	1.00					
Education								
<Secondary	84	52	1.11	0.72–1.704	0.60	0.85	0.42–01.71	0.66
≥Secondary	147	101	1.00					
Salary employment								
No	199	105	2.84	1.71–4.71	<0.001	1.55	0.55–4.34	0.41
Yes	32	48	1.00					
BF experience								
Yes	113	146	1.63	1.06–2.51	0.025	0.55	0.77–3.91	0.55
No	23	8	1.00					
Baby's age								
>2 days	93	38	2.04	1.29–3.20	0.002	1.79	0.93–3.43	0.08
1–2 days	138	115						
Baby's weight (kg)								
≥2.5	208	135	1.206	0.63–2.32	0.56	1.03	0.47–2.27	0.93
<2.5	23	18	1					
BF health education								
Yes	151	86	1.47	0.97–2.24	0.71	0.28	0.27–2.98	0.29
No	80	67	1.00					
BF demonstration								
Yes	155	91	1.42	0.93–2.17	0.10	7.20	0.73–7.45	0.09
No	75	62						
BF support								
Yes	188	106	1.94	1.2–3.12	0.007	4.45	1.95–6.12	0.008^a^
No	43	47	1					

Notes

BF: breastfeeding; BFSES: Breastfeeding Self‐Efficacy scale; SVD: spontaneous vaginal delivery.

a Statistically significant.

## 4 DISCUSSION

The study set out to determine the predictors of BFSE using the 14‐item short form of self‐efficacy scale. The scale was developed to measure the mother's confidence in her ability to breastfeed her baby. This was established among women at the postpartum unit before discharge after delivery. The BFSE determined how much effort mothers place on breastfeeding, how she will respond to any challenges and how long she will persevere breastfeeding in the face of obstacles. In this study the Cronbach's alpha of the BFSES is (0.89), this implies that the scale items reliably measured the intended factor which was the BFSE. From the studies that have been done in western countries like Iran, Australia, China and Turkey, the recent publications show strong Cronbach's alpha scores ranging from (0.83–0.93) (Oliver‐Roig et al., 2012; Otsuka et al., 2014; Zhu et al., 2014). The overall mean score of the participants on the scale was 48.65. The BFSE levels of mothers are lower compared with studies done elsewhere. In a study that was carried out among primiparas mothers in Australia that showed the mean score to be 58.32 (Keemer, 2013). Similarly, in another study conducted in Turkey among pregnant and postnatal women the median scale score was 55.8 (Tokat et al., 2010). The difference in the scores could be speculated that the Uganda mothers do not receive adequate information regarding breastfeeding from healthcare providers. Most of the mothers (61.7%) reported not to have received any teaching or demonstration on breastfeeding. In this regard, when mothers are not supported by health professionals to breastfeed, their BFSE will be low. This implies that they will not breastfeed their babies for the recommended time and the benefits from such will not be maximized.

On the other hand, this study found out that 60.2% of the women were above the cut‐off of the BFSES. The results of this study indicated that a greater number of women had a high BFSE based on the cut‐off. This implies that a greater number of women are confident in breastfeeding their babies. On the other hand, a statistically significant number of women (39.8%) had a low BFSE. This may not be surprising given the fact that women who do not exclusive breastfeed their children in Uganda is approximated to 40%. This implies that such women cannot cope with the challenges hence giving up on exclusive breast feeding. On an important note, identification of such mothers allows healthcare professionals to provide appropriate intervention to modify their attitudes and enhance their confidence to breastfeed (Zhu et al., 2014).

### 4.1 Predictors of breastfeeding self‐efficacy

At the bivariate level, maternal BFSE was associated with the mode of delivery, the relationship with the partner or presence of a spouse, employment status and breastfeeding social support. Education did not statistically predict BFSE. Participants with a higher level of education were 0.3 times less likely to have low BFSE. This could be because mothers with higher levels of education normally prefer using formula and bottle feeding due to the fact that some are working and do not have enough time to breastfeed while others have money and can afford to buy their babies formula. However in a similar study that was carried out among postnatal mothers in Spain by Oliver‐Roig and others, findings show that education level of mothers is not related with BFSE (Gerhardsson et al., 2014). The difference in the results could be due to the difference in the sample population considered, whereby the mothers selected to participate in the study were having the same education level.

Secondly, the decision time to breastfeed was also statistically significant at bivariate level. Mothers who had made a decision to breastfeed before pregnancy were 0.6 times less likely to have a low BFSE. This finding was similar to the study carried out among Chinese mothers to determine the predictors of BFSE (Zhu et al., 2014). This implies that mothers who have intentions to breastfeed will perceive themselves able to perform the act successfully then they will breastfeed for the recommended time.

In the studies that were carried out among postnatal mothers in Spain, china and Sweden reported that women who had a previous successful breastfeeding experience were more likely to have a high BFSE than new mothers (Gerhardsson et al., 2014; Oliver‐Roig et al., 2012; Zhu et al., 2014). In this study, the previous breastfeeding experience of women was not found to be a predictor of BFSE at both bivariate and multivariate analysis levels. The differing results could be partially explained by the difference in the study design that would not permit to follow up the mothers to exactly establish if mothers who had a previous breastfeeding experience could breastfeed their babies exclusively for 6 months and continue breastfeeding with complementary foods up to 2 years as recommended by the WHO (World Health Organization, 2008).

Marital relationship and social support were the factors that were found to be significantly related to BFSE. Various studies that have been carried out in countries like China and Australia show that mothers who had support from their spouses were most likely to have a high BFSE and would breastfeed their babies for the recommended time (Keemer, 2013; Zhu et al., 2014). This is because verbal persuasion from significant others such as family and health care profesionals encourage mothers to continue breastfeeding their infants amidst challenges.

### 4.2 Limitations

The study was done among mothers attending a postnatal clinic therefore we were not able to measure the BFSE of all mothers in Uganda. This implies that the results obtained in this study are not final and a clear representation of all postnatal mothers in Uganda.

## 5 CONCLUSION

The factors that were associated with BFSE include having a partner and having received breastfeeding support from the health workers after delivery. A notable number of women had a low breastfeeding confidence. This shows that such women are at risk of prematurely stopping breastfeeding. Prospective studies using the BFSES‐SF are needed to provide support for the theoretical perspective that BFSE is related to future breastfeeding behaviour. Midwives in clinical setting need to educate women on exclusive breastfeeding and support for the women to improve their BFSE.

## 6 RELEVANCE TO CLINICAL PRACTICE

The finding of the study provides a direction for midwives in maternity care in educating and supporting women about breastfeeding for the improvement of exclusive breastfeeding rates thus realization of benefits of exclusive breastfeeding.

## CONFLICT OF INTEREST

The authors declare no conflict of interest.
